# Digital Support for Daily Oral Hygiene: A Mobile Application to Improve Patients’ Adherence and Management of Periodontitis—Initial Implementation and User Feedback

**DOI:** 10.3390/dj13110520

**Published:** 2025-11-06

**Authors:** Vlad-Mihai Morariu, Andrada Soancă, Alexandra Roman, Silviu Albu, Anda Gâta, Ștefan Vesa, Petra Șurlin, Diana Tăut, Marius Negucioiu, Andreea Cândea

**Affiliations:** 1 Department of Periodontology, Applicative Periodontal Regeneration and Pediatrics Dental Medicine Research Center, Iuliu Hațieganu University of Medicine and Pharmacy Cluj-Napoca, Victor Babeș St., No. 15, 400012 Cluj-Napoca, Romania; vlad.miha.morariu@elearn.umfcluj.ro (V.-M.M.); andrada.popovici@umfcluj.ro (A.S.); andreea.ciurea@umfcluj.ro (A.C.); 2 Emergency County Clinical Hospital, Clinicilor St., No. 3-5, 400347 Cluj-Napoca, Romania; marius.negucioiu@umfcluj.ro; 3 2nd Department of Otolaryngology, Iuliu Hatieganu University of Medicine and Pharmacy, Republicii St., No. 18-20, 400015 Cluj-Napoca, Romania; silviualbu63@elearn.umfcluj.ro (S.A.); gataanda@elearn.umfcluj.ro (A.G.); 4 Department of Pharmacology, Toxicology and Clinical Pharmacology, Iuliu Hațieganu University of Medicine and Pharmacy, Marinescu St., No. 23, 400337 Cluj-Napoca, Romania; 5 Department of Periodontology, Research Center of Periodontal-Systemic Implications, Faculty of Dental Medicine, University of Medicine and Pharmacy, Petru Rares St., No. 2, 200349 Craiova, Romania; petra.surlin@umfcv.ro; 6 Department of Psychology, Babeș-Bolyai University, Republicii St., No. 37, 400015 Cluj-Napoca, Romania; dianataut@psychology.ro; 7 Department of Prosthodontics, Faculty of Dental Medicine, Iuliu Hațieganu University of Medicine and Pharmacy Cluj-Napoca, Clinicilor St., No. 32, 400006 Cluj-Napoca, Romania

**Keywords:** mHealth, mobile application, periodontitis, oral hygiene, adherence, education

## Abstract

**Background:** Maintaining daily optimal dental hygiene, especially in medically vulnerable patients with periodontitis, remains challenging in dental practice. Mobile apps and other digital tools might offer useful support alongside traditional advice. **Objectives:** This study aimed to develop a mobile health app, *PerioSupportPro*, that helps patients improve their daily plaque control habits. It also reports on the pilot testing of the app’s usability and users’ perception in a small patient group. **Methods:** The app was created by a mixed team including periodontists, psychologists, developers, and data protection specialists. The first version included reminders, gamified elements, video tutorials, and motivational messages. After internal testing, a group of 18 patients tested the app and completed a feedback questionnaire that assessed usability (Q3–Q5), educational impact (Q6–Q8), motivation (Q9–Q11), and overall satisfaction (Q12–Q14). Cronbach’s alpha was used to check internal consistency, and non-parametric tests were applied for basic statistical comparisons. **Results:** The motivation section of the questionnaire showed acceptable consistency (α = 0.784), while usability and educational impact had lower values (α = 0.418 and 0.438). No clear differences were found between age groups. Satisfaction was positively associated with reminders and motivational items. Most appreciated features included reminders, the simple interface, and short videos. Based on the input provided by the questionnaire, a few improvements were made, and a second version of the app was prepared. **Conclusions:** Early user responses show that *PerioSupportPro* may help motivate and guide patients in their oral hygiene routine. While still in an early phase, the app seems well-received and ready for future clinical validation with more users.

## 1. Introduction

Health mobile applications (mHealth apps) have emerged as a logical consequence of digital advancements in mobile applications in all fields, offering accessibility, real-time feedback, emotional engagement, and instant gratification [1,2]. mHealth apps are used for remote consultation, disease diagnosis, behavior modifications [1], decision-making [3], management of chronic diseases, and promoting healthy living [4].

By 2015, over 500 million users had adopted mHealth tools [5], and as of 2017, more than 325,000 apps were available [6]. A total of 1075 oral hygiene apps were available as of 2018 [7]. While mHealth use in oral health is expanding—especially among children and adolescents [1]—the quality and focus of such apps remain inconsistent [8].

Periodontitis affects nearly half of the global population, with severe forms impacting over 10% of individuals worldwide [9]. In medically vulnerable patients, the prevalence of periodontitis and plaque accumulation is significantly higher [10,11]. Daily oral hygiene remains essential for primary and secondary prevention of both caries and periodontitis in adults and children [12]. Still, adherence to standard routines is low, with a third of men brushing less than twice daily and 59% of women skipping bedtime brushing [13].

Beyond traditional instruction, there is a need for innovative tools adapted to contemporary lifestyles to help patients keep a long-term optimal oral care routine. Apps like *Brush DJ* [14], *My PerioCare* [15], *MyPerioHealth* [16], *AI-MST* [17], and other apps (Supplementary Materials, Table S1: Identified mobile applications (apps) to improve oral hygiene) [18,19,20,21,22,23] illustrate varied approaches to support oral care. Meta-analyses confirm their short-term efficacy in reducing plaque and improving gingival health through motivation and habit-building [1,24,25,26]. However, factors like cost, language, and copyright remain barriers to the widespread use of oral hygiene apps [27].

Based on these empirical considerations, the aim of this study was to develop an mHealth app named *PerioSupportPro* to sustain dental patients, particularly periodontitis patients with medical vulnerabilities, in performing adequate personal oral hygiene. Also, this study aimed to validate a second variant of the *PerioSupportPro* app through pilot clinical usability testing.

## 2. Materials and Methods

### 2.1. Study Design

The study was initiated by a periodontology team that aimed to develop *PerioSupportPro* as a user-friendly mHealth app that reminds Romanian patients to clean their teeth daily and teaches them efficient cleaning methods. The target audience is represented mostly by patients with periodontal-systemic problems, but also general dental patients or the general public.

The development of *PerioSupportPro* was carried out in close collaboration with an interdisciplinary team. *PerioSupportPro* was tested for usability and engagement among patients by using an AGILE iterative design approach.

The ethical approvals were obtained from the Ethics Committees of Iuliu Hatieganu University of Medicine and Pharmacy Cluj-Napoca (118/4.06.2024) and County Emergency Hospital Cluj-Napoca (52952/9.11.2023).

### 2.2. PerioSupportPro Mobile App Development

**Figure 1:**
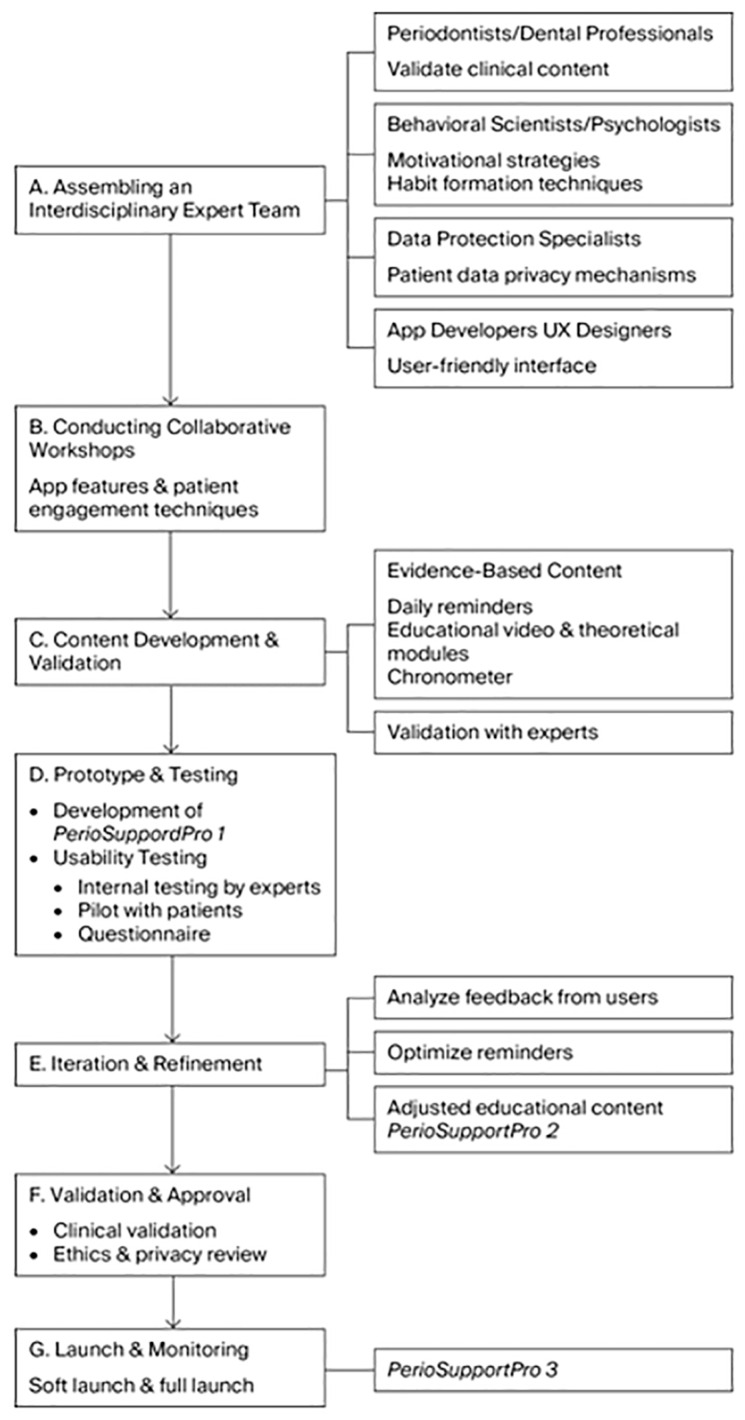
Flowchart of the developing steps of the *PerioSupportPro* app.

The flowchart outlining the development steps of the *PerioSupportPro* app is shown in Figure 1, and detailed developmental steps of the *PerioSupportPro* app are provided in Supplementary Materials, Table S2: Detailed steps of *PerioSupportPro* App development.

**A. Assembling an interdisciplinary expert team** ensured both scientific and technical accuracy.

**A(1) Periodontists** designed and approved the clinical content, including oral cleaning techniques, recommended frequency, and educational material.

**A(2) Behavioral scientists/psychologists** developed motivational strategies and habit-formation techniques. The app incorporates both intrinsic and extrinsic motivators as integrated rewards (such as badges and level-progression feedback). Push notifications and reminders encourage users to follow daily routines, while rewards and progress tracking support long-term habit formation. The information is presented in simple, clear language with easy-to-follow visual steps. **Nudges** (small prompts) are also included to stimulate daily activity (Supplementary Materials, Table S3: Encouraging prompts and educational content of *PerioSupportPro* app).

**A(3) Data protection specialists** ensured compliance with privacy regulations such as the **GDPR (General Data Protection Regulation)** and **HIPAA (Health Insurance Portability and Accountability Act)**. They oversaw data security, encryption protocols, risk assessments, and contingency plans for potential data breaches.

**A(4) App developers and UX designers** focused on creating a user-friendly interface that is intuitive and accessible for patients.

**B. Conducting collaborative workshops** with all team members facilitated agreement on the app’s core functions and understandability, mapping of patient journeys, and discussions about patient needs and potential challenges.

**C. Content Development and Validation.**

The team developed content based on current evidence, including **daily motivational reminders** (varied to avoid monotony; Supplementary Materials, Table S3: Encouraging prompts and educational content of *PerioSupportPro* app), **short educational modules**, and **more detailed theoretical material** (also in Supplementary Materials, Table S3: Encouraging prompts and educational content of *PerioSupportPro* app). The content was reviewed and approved by both periodontists and behavioral experts.

**D. Prototype and Testing.**

**D(1)** A basic working version of the app (**Minimum Viable Product, MVP**) was built with key features for testing: an easy-to-use interface, secure user authentication (personal access code), daily reminders, progress tracking, a chronometer, and **gamification elements** (e.g., medals awarded when users view videos for at least six seconds). Initial content included videos and text on brushing, interdental cleaning, gingivitis, and periodontitis, as well as motivational messages. The app also tracks **engagement metrics** such as frequency of use, session duration, notification interactions, and user satisfaction.

**D(2) Application Creation.** The app was developed in **Flutter 3.24.x (using the Dart 3.4.x SDK)** enabling efficient cross-platform development for both Android and iOS with a single codebase, thereby saving time and costs while maintaining near-native performance. The design was initially created in **Figma** to allow easy testing and modification of layouts and features before coding. **Firebase (Google)** was used to manage notifications, behavior tracking, performance monitoring, and secure data storage. The **minimum supported platform versions** were **Android 6.0 (API level 23)** and **iOS 13.0**.These steps resulted in the first working version of the *PerioSupportPro* app.

**D(3) Usability testing** involved two phases. First, internal testing by experts (periodontists, UX designers, and psychologists) focused on refining content, interface, and motivational tools was carried out. Subsequently, a small cohort of participants tested the app and provided feedback through a questionnaire on usability, clarity of the wording, the friendliness of the interface, and the usefulness of the content (see Section 2.3). Participants were recruited consecutively from patients scheduled for prophylactic procedures at the Department of Periodontology, from Iuliu Hatieganu University of Medicine and Pharmacy Cluj-Napoca and County Emergency Hospital Cluj-Napoca. No formal periodontal examination was performed. In line with the exploratory design, no exclusion criteria were applied, and all patients who consented during the recruitment period were included. The recruitment took place over a short time window of two weeks, during which a total of 18 patients agreed to participate and completed the usability questionnaire; no participants declined or were excluded at any stage. The small consecutive sample (*N* = 18) was intentionally selected for qualitative exploration of usability and comprehension. This preliminary phase aimed to refine the app’s content and functionality before a larger-scale validation study.

**E. Iteration and Refinement.**

Based on patient questionnaire feedback, the app was improved. Experts adjusted the timing and tone of reminders, and the length of educational materials was modified for greater efficiency. These revisions produced the **second version** of the *PerioSupportPro* app.

**F. Validation and Approval.**

Clinical testing through a randomized trial to evaluate the app’s impact on oral hygiene habits has not yet been conducted. A **third version** of the app will be developed after clinical validation.

Privacy and data protection procedures followed ethical standards. The app collects only phone numbers for user identification, linked to private access codes. The full list of patient identities is securely held exclusively by the study coordinator (V.M.M). All usage data are stored securely and linked only to access codes, without personal identifiers.

**G. Launch and Monitoring.**

**G(1) A soft launch** tests the app under real-world conditions with a small group (10 dentists and 10 patients). This phase evaluates functionality across different devices, stability, user response outside the study setting, and technical performance (server load, notifications, and app crashes).

**H(2) A full launch** is planned to expand access to a wider audience—periodontists, general dentists, patients, and even medical doctors managing patients with systemic conditions related to periodontal disease. The overall data will be used to inform future updates.

After launch, app performance will be monitored over time. Long-term data will support improvements to both motivational strategies and educational content. **Community engagement**—including user stories, reviews, and discussion spaces—will also be encouraged to foster support and sustained user activity.

### 2.3. Questionnaire for Feedback on Usability and Engagement

A cross-sectional qualitative user perception questionnaire developed to examine understandability, as well as the experiences and beliefs of people using the *PerioSupportApp*, was created using Google Forms. The questionnaire targeted 4 domains as follows: section 1—User Profile investigating user age (Q1) and known periodontitis diagnosis (Q2); section 2—App Usability (Q3–Q5); section 3—Educational and Behavioral Impact (Q6–Q8); section 4—Motivation and Engagement (Q9–Q11); and section 5—Overall Satisfaction and Suggestions appreciating overall satisfaction (Q12), positive features of the app (Q13), and suggestions for improvements (Q14). Items Q3–Q12 used an appreciation rating scale from 1 to 4 (score 1 is the worst experience and 4 is the most positive one).

The questionnaire was piloted, then updated from the feedback and piloted again before final distribution. The questionnaire can be found at the following link: (https://docs.google.com/forms/d/e/1FAIpQLSeLXSKKCmVzxPkV7We_9iWWdnPKQTNrX0jRUAUh6QTxOsnU0Q/viewform?usp=header, accessed on 4 July 2025) (Table 1).

### 2.4. Statistical Analysis

Statistical analysis was performed using the MedCalc^®^ Statistical Software version 23.1.6 (MedCalc Software Ltd., Ostend, Belgium; https://www.medcalc.org; 2025).

Descriptive statistics (frequencies, percentages, median, and 25 and 75 percentiles) were used to summarize participant characteristics and responses to the *PerioSupportPro* app evaluation questionnaire. To assess the internal consistency of grouped Likert-scale items (section 1—App Usability Q3–Q5; section 2—Educational/Behavioral Impact Q6–Q8; section 3—Motivation Q9–Q11), Cronbach’s alpha was calculated.

Group comparisons between patient subgroups based on age (≤30 and >30) were performed using the Mann–Whitney test to examine differences in perceived usability, motivation, and satisfaction scores across the two groups. Correlations between ordinal responses (e.g., usability and satisfaction) were explored using Spearman’s rank correlation coefficient. We examined differences in usability predictors between moderately and highly satisfied users (Q12 = 3 and 4) through the Mann–Whitney test.

Statistical significance was considered at a *p* value < 0.05.

## 3. Results

This study describes the development and initial implementation of *PerioSupportPro*, an mHealth educational application designed primarily to support medically vulnerable patients with periodontitis, but also adaptable for broader populations seeking to improve their daily dental plaque control habits.

**Figure 2:**
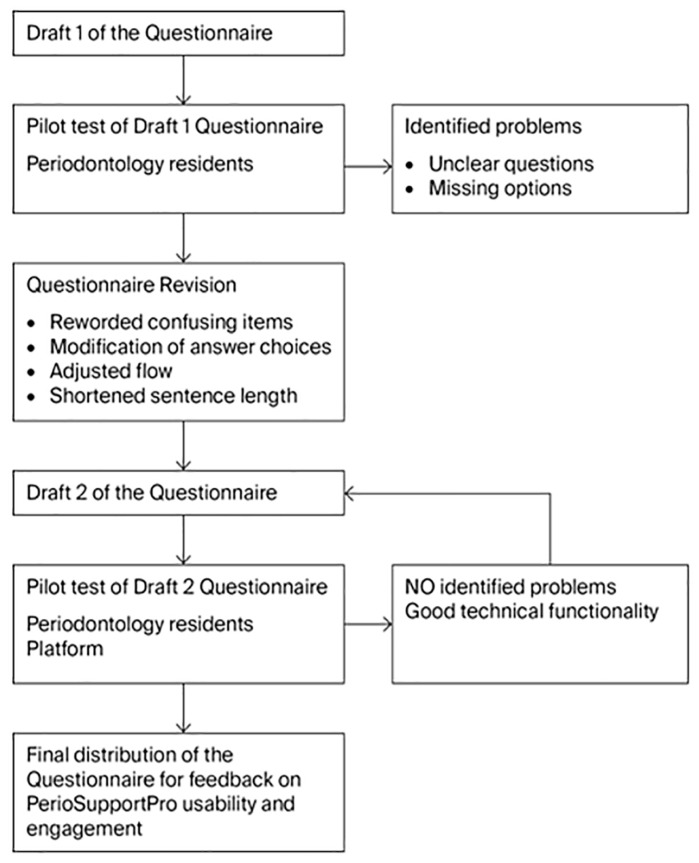
Validation and refinement process of the questionnaire for *PerioSupportPro* usability testing.

The first version of the *PerioSupportPro* app was developed and then tested for internal usability and updated by experts. This improved first version of *PerioSupportPro* was used for pilot testing on a small cohort of 18 subjects. Participants’ experiences were collected using a structured questionnaire that was carefully piloted and refined prior to distribution (Figure 2).

Internal consistency values for the App Usability (α = 0.418) and Educational and Behavioral Impact (α = 0.438) sections of the *PerioSupportPro* questionnaire were modest as opposed to the value obtained for the Motivation and Engagement section, which showed acceptable consistency (α = 0.784).

Participants were divided into two groups based on age (Q1), and the related ordinal responses were analyzed accordingly (Table 2). The periodontal status item (Q2) was not analyzed due to the small number of responses. Comparisons between age groups (18–30 vs. 31–60 years) revealed no statistically significant differences in perceived usability, educational impact, motivation, or satisfaction. Median scores were generally similar across age groups, with slightly higher ratings for interdental tool use among older participants, although this difference did not reach statistical significance (*p* = 0.149).

Spearman’s rank correlation analysis showed significant positive associations between overall satisfaction (Q12) and two items from the App Usability section (Q4 and Q5), as well as all items from the Motivation and Engagement section (Q9–Q11). Moderate but non-significant correlations were observed between Q12 and Q3 (App Usability) and all items from the Educational and Behavioral Impact section. These findings are summarized in Table 3.

Due to the small sample size (*N* = 18), inferential results are interpreted with caution and primarily serve to identify potential trends to be validated in future studies.

Examining differences in usability predictors between moderately and highly satisfied users (Table 4) revealed that participants who reported higher overall satisfaction also tended to rate the usefulness of reminders significantly higher than those with medium satisfaction (*p* = 0.012). Differences in perceived ease of use (Q3) and visual appeal (Q5) did not reach statistical significance.

Six participants did not provide comments on the most liked features of the app (Q13). Among the others, reminders, the user-friendly interface, explanatory videos, and rewards were mentioned positively by 5, 4, 3, and 2 participants, respectively.

One of the educational explanations was seen as too technical (Q14) by a participant.

Based on the feedback from the questionnaire, adjustments were made, leading to the second version of the *PerioSupportPro* app (https://apps.apple.com/app/periosupportpro/id6738766090, (accessed on 4 July 2025), https://play.google.com/store/apps/details?id=com.periosupportpro.android&hl=en, (accessed on 4 July 2025)).

Some features of *PerioSupportPro 2* are revealed in the Supplementary Materials, Figure S1: The second version of the *PerioSupportPro* app.

## 4. Discussion

Our team followed several key development steps in building the *PerioSupportPro* app, aiming to create a solid and evidence-based tool to support better oral hygiene habits. The first prototype of *PerioSupportPro* was developed, tested for usability, and adjusted based on user feedback, resulting in a second version that is now ready for further clinical validation and launch.

The *PerioSupportPro* app was piloted by a small cohort of patients using a structured questionnaire covering four domains. The objective of the pilot study was not statistical power or outcome validation, but the appreciation of the clarity and accessibility of the wording, the friendliness of the interface, and the usefulness of the content. Others assessed the app’s acceptability through a feedback interview [28]. The usability questionnaire was employed as part of a formative, developmental evaluation to refine the app’s content and interface. This exploratory phase focused on clarity, comprehensibility, and user experience, not on psychometric or outcome validation. Therefore, a large sample size or adherence to STROBE reporting standards was not applicable at this stage.

The Motivation and Engagement section showed good internal consistency (α = 0.784), indicating stronger coherence and better acceptance by participants. In contrast, lower internal consistency values were observed in two other sections—App Usability (α = 0.418) and Educational and Behavioral Impact (α = 0.438), possibly due to the limited number of items included in each or to the multifaceted nature of the evaluated constructs. Although both sections included items targeting different dimensions, none of them behaved as a unidimensional scale. The shortness of the questionnaire might be considered a limitation of the study. At the same time, a longer questionnaire would not have been practical, so we chose to keep it brief. Still, the responses provided useful insights into how users experienced the app and what benefits they perceived.

The questionnaire was similarly received by participants regardless of age (Table 2).

The analysis showed that Q12 (overall satisfaction) was significantly and positively correlated with the perceived usefulness of reminders (Q4) (*p* = 0.003), visual appeal/user-friendliness (Q5) (*p* = 0.020), and all motivation and engagement items (motivation from gratifications, Q9, or educational materials, Q10, and likelihood of continued use, Q11).

*PerioSupportPro* was developed in response to the well-known limitations of conventional motivational and instructional strategies, which have often proven insufficient in promoting long-term improvements in personal oral hygiene among certain patient groups [10,11,13]. In contrast, mHealth approaches have shown better results in improving motivation, adherence to oral hygiene routines [1,24,25,29], and state of mind during brushing [14].

Among the most appreciated features of *PerioSupportPro* were the reminders, educational videos, and the reward system—qualities that have also been reported to positively influence user engagement with mHealth apps in other studies [28,30]. Reminders are a central component of many oral health apps: by mid-2014, over one million reminders had already been sent to users of the *Brush DJ* app [14]. In fact, the initial idea for *PerioSupportPro* was actually inspired by Duolingo’s approach to reinforcement.

Users appreciated the educational videos, known as important elements for improving brushing skills and understanding [14,31]. Based on open-ended feedback from the questionnaire, we simplified the educational content, recognizing its role in addressing patients’ questions that their dentist may not have had time to fully explain [14]. This allows clinicians to save time during routine visits and focus more on clinical interventions [14].

The interface itself was praised by both testers and patients, confirming how vital usability is in mHealth design [30].

Short-term use of the *PerioSupportPro* app induced enthusiastic appreciations, but future steps for implementing and validating *PerioSupportPro* would clarify factors that influence adherence to it. Data on long-term adherence to oral health apps is limited [30]. Personal support, complementary to digital intervention, has been identified as mandatory [30], but it is certainly insufficient to sustain a long-term maintenance program. We are witnessing a paradigm shift driven by a generation raised with mobile technology—often spending more time on phones than with family or work. This enables health interventions anytime and anywhere [14]. The pandemic further accelerated digital tools’ integration in healthcare and education [14,32].

*PerioSupportPro* is developed to align with the ongoing paradigm shift toward mobile health tools [2]. One of our app’s clear strengths is its accessibility: it is completely free, has no ads or in-app purchases, is compatible with any kind of toothbrush, and delivers education grounded in evidence-based content. To the best of our knowledge, it is also the first Romanian app of its kind currently available. An additional benefit is its potential to reduce disparities in access to proper personal oral care, especially in rural communities [33].

We perceived some general limitations that apply to oral mHealth apps, such as cost-related concerns when compared to traditional motivational methods [14], along with linguistic and educational barriers. To address the latter, we designed the app to be as simple as possible, keeping in mind that health literacy greatly affects people’s ability to access, understand, and apply health-related information [28]. It is worth noting that health literacy remains low or marginal in both the U.S. and EU populations [28,34,35]. From this perspective, one limitation of our study is that our app testers had a generally high level of education.

The *PerioSupportPro* app has a limited educational content linked to the small budget allocated for development. By contrast, other more complex apps include external resources such as links to NHS Smokefree and Alcohol support websites [36,37], or animated oral hygiene videos hosted on YouTube [14].

## 5. Conclusions

The pilot implementation of *PerioSupportPro* highlighted strong user motivation and engagement, with this domain showing the highest internal consistency (α = 0.784), suggesting that the app’s motivational strategies were well-received by participants. The testers of *PerioSupportPro* generally reported positive experiences, with no significant differences observed across age groups.

Questionnaire feedback indicated that reminders, user interface, and video content were among the most valued features of the app.

Based on user input, educational content was refined, resulting in a second, improved version of the *PerioSupportPro* app ready for further clinical validation.

## Figures and Tables

**Table 1:** *PerioSupportPro*—app evaluation questionnaire.

**Section 1—User Profile**	**Section 3—Educational and Behavioral Impact**	**Section 5—Overall Satisfaction and Suggestions**
Q1. What is your age? ☐ 18–30 ☐ 31–50 ☐ 51–60 ☐ over 60 Q2. Do you currently have a diagnosed periodontal condition? ☐ No ☐ Not sure ☐ Yes, mild ☐ Yes, moderate/severe	Q6. To what extent did the educational videos improve your understanding of oral hygiene techniques? ☐ Not at all ☐ Slightly ☐ Moderately ☐ Significantly Q7. Have you improved your oral hygiene habits as a result of using the app? ☐ Not at all ☐ Slightly ☐ Moderately ☐ Significantly Q8. How often do you use interdental hygiene tools after being guided by the app? ☐ Never ☐ Occasionally ☐ Frequently ☐ Daily	Q12. Overall, how satisfied are you with the app? ☐ Not at all satisfied ☐ Slightly satisfied ☐ Moderately satisfied ☐ Very satisfied Q13. What features did you like most? [Open-ended] Q14. What would you suggest to improve the app? [Open-ended]
**Section 2—App Usability**	**Section 4—Motivation and Engagement**	
Q3. How easy was it to use the app? ☐ Very difficult ☐ Somewhat difficult ☐ Somewhat easy ☐ Very easy Q4. How useful were the daily reminders in maintaining your brushing routine? ☐ Not useful at all ☐ Slightly useful ☐ Moderately useful ☐ Very useful Q5. How visually appealing and user-friendly was the app interface? ☐ Not at all ☐ Slightly ☐ Moderately ☐ Significantly	Q9. To what extent did the gratifications (rewards, feedback, emojis, badges etc.) motivate you to keep using the app? ☐ Not at all ☐ Slightly ☐ Moderately ☐ Strongly Q10. To what extent did the educational materials (videos, tips, written content) motivate you to improve your oral hygiene routine? ☐ Not at all ☐ Slightly ☐ Moderately ☐ Strongly Q11. How likely are you to continue using this app regularly? ☐ Not likely at all ☐ Slightly likely ☐ Moderately likely ☐ Very likely	

Abbreviations: Q, question.

**Table 2:** Comparison of app evaluation scores by age groups (18–30 vs. 31–60 years).

Section	Question *	Median (First to the Third Quartile) 18–30 yrs, N = 7 (38.9%)	Median (First to the Third Quartile) 31–60 yrs, N = 11 (61.1%)	*p*-Value
Section 2—App Usability	Q3. Easy to use	4.00 (3; 4)	4.00 (3; 4)	0.661
	Q4. Usefulness of reminders	4.00 (3; 4)	4.00 (3; 4)	0.789
	Q5. Visual appeal/user friendliness	4.00 (3; 4)	4.00 (3; 4)	0.913
Section 3—Educational and Behavioral Impact	Q6. Improved understanding from videos	4.00 (3; 4)	4.00 (4; 4)	0.708
	Q7. Improved hygiene habits	3.00 (3; 4)	3.00 (3; 4)	0.515
	Q8. Improved use of interdental tools	2.00 (2; 3)	3.00 (2; 4)	0.149
Section 4—Motivation and Engagement	Q9. Motivation from gratifications	4.00 (3; 4)	3.00 (3; 4)	0.550
	Q10. Motivation from educational materials	4.00 (3; 4)	3.00 (3; 4)	0.638
	Q11. Likelihood of continued use	4.00 (2; 4)	4.00 (3; 4)	0.497
Section 5–Overall Satisfaction and Suggestions	Q12. Overall satisfaction	4.00 (3; 4)	4.00 (3; 4)	0.533
	Q13. Positive features [open-ended]	-	-	-
	Q14. Suggestions for improvements [open-ended]	-	-	-

Abbreviations: Q, question. Note: * the full questions are found in the Supplementary Materials for the *PerioSupportPro* questionnaire.

**Table 3:** Spearman’s Rank correlation between app experience items and overall satisfaction (Q12).

Section	Item	Spearman ρ	*p*-Value
Section 2—App Usability	Q3. Easy to use	0.433	0.073
	Q4. Usefulness of reminders	0.666	0.003 *
	Q5. Visual appeal/user-friendliness	0.541	0.020 *
Section 3—Educational and Behavioral Impact	Q6. Improved understanding from videos	0.351	0.153
	Q7. Improved hygiene habits	0.430	0.075
	Q8. Improved use of interdental tools	0.444	0.065
Section 4—Motivation and Engagement	Q9. Motivation from gratifications	0.636	0.005 *
	Q10. Motivation from educational materials	0.531	0.023 *
	Q11. Likelihood of continued use	0.750	0.000 *

Abbreviations: Q, question. Note: * Significance threshold at *p* < 0.05.

**Table 4:** Comparison of usability scores by satisfaction level (Q12) (Q12 = 3 vs. Q12 = 4).

Usability Item	Median (First to the Third Quartile) (Q12 = 3)	Median (First to the Third Quartile) (Q12 = 4)	*p*-Value
Q3. Easy to use	3.00 (3.00; 3.75)	4.00 (3.75; 4.00)	0.180
Q4. Usefulness of reminders	3.00 (3.00; 3.00)	4.00 (3.75; 4.00)	**0.012** *
Q5. Visual appeal/user-friendliness	3.00 (2.25; 3.75)	4.00 (3.75; 4.00)	0.130

Abbreviations: Q, question. Note: * Significance threshold at *p* < 0.05.

## Data Availability

The original contributions presented in this study are included in the article/Supplementary Material. Further inquiries can be directed to the corresponding author.

## Supplementary Materials

The following supporting information can be downloaded at https://www.mdpi.com/article/10.3390/dj13110520/s1: Figure S1: The second version of the *PerioSupportPro* app; Table S1: Identified mobile applications (apps) to improve oral hygiene; Table S2: Detailed steps of *PerioSupportPro* App development; Table S3: Encouraging prompts and educational content of *PerioSupportPro* app.
