# Supplementary material for: Digital Support for Daily Oral Hygiene: A Mobile Application to Improve Patients’ Adherence and Management of Periodontitis—Initial Implementation and User Feedback
Source: Dent J (Basel). 2025 Nov 6;13(11):520. doi: 10.3390/dj13110520 (PMC12651269; doi:10.3390/dj13110520)
Supplement: Supplementary file 1 [file dentistry-13-00520-s001.zip › dentistry-3886511-supplementary.pdf]

Supplementary Materials_Morariu V et al_ Digital Support for Daily Oral Hygiene: A Mobile Application to Improve Patients' Adherence and Management of Periodontitis - Initial Implementation and User Feedback

Supplementary material - Table S1. Identified mobile applications (apps) to improve oral hygiene

Mobile App	Reference/App link*	Objectives and functions
AI-MST/App Architecture	Li et al.,2024	Associated with an Ai-driven toothbrush. Provides real-time feedback to equalize the brushing time in the mouth, to monitor brushing pressure, to highlight neglected areas
Brush DJ app	Underwood et al.2015<br>https://play.google.com/store/apps/details?id=uk.co.appware.brushdj&hl=en	Aims to implement a tooth-brushing hygiene routine by playing music.
Colgate Connect	Tiffany et al.2018<br>https://connectsupport.colgate.com/hc/en-us/categories/4417249330450-Colgate-Connect-app	Aims to improve the quality of brushing by increasing the brushing time and areas. Proposes a personalized follow-up by connecting the electric toothbrush to the app.
iGAM	Tobias et al.2020, 2021	Focuses on periodontal health and improves the information flow between dentists and patients.<br>Monitors gingivitis using self-photography
Kolibree	Tiffany et al.2018<br>Carrouel et al. 2022<br>https://www.kolibree.com/en	Increases brushing quality by extending time and covered areas, with personalized follow-up via a connected electric toothbrush.
My PerioCare	Bigot C, EFP first prize 2023<br>https://www.efp.org/publications-hub/an-app-to-help-dentists-and-patients-better-manage-periodontal-care/	Assists both dentists and patients in managing periodontal care.
MyPerioHealth	Donofrio J. 2022<br>https://www.myperiohealth.com/	Allows patients to discover the extent of their periodontitis based on the American Academy of Periodontology Staging and Grading Periodontitis Guidelines
Oralytics	Trella et al.2025<br>https://play.google.com/store/apps/details?id=edu.ucla.oarc.oralytics	Utilizes a reinforcement learning algorithm to deliver personalized prompts at optimal times when they are likely to be effective, encouraging oral self-care behaviors
OSCA (Oral Self-Care App)	Chang et al.2019	Assists periodontitis patients in improving oral hygiene through daily self-care routines. It leads to significant improvements in oral hygiene behaviors and status.
PerioUICare	Purba et al.,2024<br>Hartono et al. 2024	Provides educational content and reminder messages for gingivitis and periodontitis patients, with demonstrated clinical benefits in hygiene indices<br>Developed by Universitas Indonesia, Jakarta, Indonesia
Preventeeth	Carrouel et al. 2022	Teaches proper dental care and brushing

	https://apkpure.com/preventeeth/com.preventeeth/download/1.8	techniques, offers personalized learning, exercises, and scores to assess dental risk.
Santé Orale – SOHDEV app	Carrouel et al. 2022<br>https://mapetitefeeautiste.com/sante-orale-sohdev/<br>https://apps.apple.com/us/app/sant%C3%A9-orale-sohdev/id1122579641	Helps people with autism build daily brushing habits and prepare for dental visits, created by the SOHDEV (Sante orale, handicap, dependance et vulnerabilite) association.
Text2Floss	Hashemian et al.2014<br>https://texttofloss.com/<br>https://apps.apple.com/us/app/text2floss/id797806327<br>https://play.google.com/store/apps/details?id=com.<br>globalsmiles.text2floss	Increases flossing behavior and oral health knowledge.<br>Induces healthier oral-related mothers' behaviors with respect to their children
Truthbrush	Carrouel et al. 2022<br>https://www.truthbrush.com/#/	Tracks brushing habits with detailed graphs, distinguishes actions like brushing and cleaning, and offers goals, rewards, alerts, and dentist data sharing when paired with the Toothbrush device.

* *if available*

Supplementary material - Table S2. Detailed steps of *PerioSupportPro* App development

Step	Step (continuation)
**A. Assembling an *Interdisciplinary Expert Team*** ensured both scientific and technical accuracy.<br>A1)*Periodontists* designed and approved clinical content such as oral cleaning techniques, recommended frequency, and educational information.<br>A2)*Behavioral Scientists/Psychologists* worked on motivational strategies and habit formation techniques. The app includes both intrinsic and extrinsic motivators as integrated rewards (like badges, level progression feed-back). Push notifications and reminders help users to follow routines, while rewards and progress tracking help with long-term habit formation. The information is kept simple, with clear language and easy-to-follow visual steps. Nudges (small prompts) are also used to encourage daily activity.<br>A3)*Data Protection Specialists* helped make sure the app followed privacy rules like GDPR (General Data Protection Regulation) and HIPAA (Health Insurance Portability and Accountability Act) rules. They took care of things like keeping user info safe, using encryption, checking for risks, and having a plan in case something goes wrong with the data.<br>A4)*App Developers & UX Designers* focused on creating an interface that is easy to use and understandable for patients.<br>**B. Conducting Collaborative Workshops** with all team members helped to agree on the app's main functions, map patient journeys, as well as discuss patient needs and potential challenges.<br>**C. Content Development & Validation.**<br>The team developed content based on current evidence including daily motivational reminders (varied to avoid boredom), short educational modules, and more detailed theoretical educational material. The content was reviewed and approved by periodontists and behavioral experts.<br>**D. Prototype & Testing.**<br>D1) A basic working version of the app (Minimum Viable Product MVP) was built, including key features for testing: easy-to-use interface, secure used authentication (personal code), daily reminders, progress tracking, a chronometer, and gamification (medals given when users open videos for at least 6 seconds). Initial content included videos and texts about brushing, interdental cleaning, gingivitis, and periodontitis, along with motivational messages. The app also tracks engagement metrics: how often it's used,	D2)Application creation. The app was developed in Flutter (Dart language), which allowed fast building for both Android and iOS using a single codebase, thereby saving time and costs while maintaining performance very close to native solutions. The design was first made in Figma in order to test and change more easily layout and features before coding. Firebase (by Google) was used to handle notifications, behavior tracking, performance monitoring, and secure data storage. This led to the first working version of the *PerioSupportPro* app.<br>D3)Usability testing followed two phases First, internal testing was done by experts (periodontists, UX designers, and psychologists) to improve content, interface, and motivational tools. Then, a small cohort of patients tested the app and sent feedback through a questionnaire about usability and engagement to oral hygiene habits (see section 2.3).<br>**E. Iteration & Refinement.**<br>After the first round of usability testing, the app was improved based on feedback provided by app users through questionnaires. Experts made changes to reminder timing and tone. The length of educational materials was also adjusted to improve efficiency. These updates led to the second version of the *PerioSupportPro* app.<br>**F. Validation & Approval.**<br>Clinical testing of the app's effect on oral hygiene habits has not yet been done. The third version of the app will be created after such validation.<br>Privacy and data protection steps followed ethical rules. The app only uses phone numbers for user identification, linked to a private access code. Only the study coordinator (V.M.) keeps the full list of patient identities. All usage data is stored securely and linked to access codes only, without personal details.<br>**G. Launch & Monitoring.** G1)Soft Launch tests the app for real-world conditions with a small group (10 dentists and 10 patients). This phase checks how the app works across different phones, how stable it is, and how users respond outside the study setting, as well as technical issues (server performance, notifications, app crashes).<br>H2)Full Launch is planned to reach more users—periodontists, general dentists, patients, and even medical doctors who manage patients with

session time, notification interactions, and user satisfaction.	systemic conditions linked to periodontal diseases and the overall date will be used for further up-dates.<br>After launch, app performance will be monitored over time. Long-term data will help improve both motivation strategies and education content. Community engagement (like user stories, reviews, and discussion spaces) will also be encouraged to support users and keep them active.

Supplementary material - Table S3. Encouraging prompts and educational content of *PerioSupportPro* app

Prompts encouraging daily routine	
	○ A healthy smile starts with a simple routine.<br>○ General health begins with healthy gums.<br>○ I'm kindly reminding you to take time for your evening brushing.<br>○ It's time to think about your oral health.<br>○ Just 2 minutes to protect your teeth in the long run.<br>○ Prevention is easier than treatment.<br>○ Brush your teeth today and thank yourself tomorrow.<br>○ Evening brushing is the key to a healthy smile.<br>○ A small effort today, a big difference tomorrow.<br>○Your teeth need you.
**Educational Content**	
Gingivitis	1.Gingivitis is an inflammation of the gums, caused by the accumulation of bacteria on the teeth due to poor dental hygiene. 2. Bacteria in the mouth are normal and necessary for health, but if they multiply too much, they can lead to inflammation of the gums. 3. Gingivitis can be completely cured if treated in time, but if ignored, it can develop into periodontitis, a more serious condition. 4.Treatment of gingivitis involves better dental hygiene and professional teeth cleaning by a dentist.
Periodontitis	1.Periodontitis is a chronic infection caused by excessive accumulation of bacteria on the teeth, which in certain conditions initiates the destruction of tooth supporting tissues including bone. 2. Incorrect tooth brushing allows dental plaque to accumulate, which leads to inflammation of the gums and, over time, the destruction of the periodontium. Diseases such as diabetes and external factors such as smoking increase the risk of developing periodontitis. 3.In the early stages, periodontitis has no obvious symptoms, which is why it is important to go for regular dental check-ups. In more advanced stages, bleeding gums, gum retraction, teeth that appear longer, bone loss, mobility and tooth displacement occur, which becomes worrying for the patient and the doctor. 4.Treatment of periodontitis consists of procedures that eliminate the local infection. The earlier it is discovered, the more effective the treatment is. 5. People who have had periodontitis must receive special care for the rest of their lives, even if the disease has been treated.
Periodontitis and general health	1.Periodontitis not only affects the gums and bone that support the teeth, but can also affect the health of the entire body, negatively impacting pregnancy, diabetes, the heart, the brain, and autoimmune diseases such as rheumatoid arthritis. 2. In severe cases, periodontitis can increase the risk of heart attack or stroke and can lead to a higher death rate. 3. It is essential to treat periodontitis as soon as possible to protect the general health of the body. 4. Bacteria from gum infections and the constant inflammation caused by periodontitis can enter

	the bloodstream, which explains the link between this disease and other health problems.

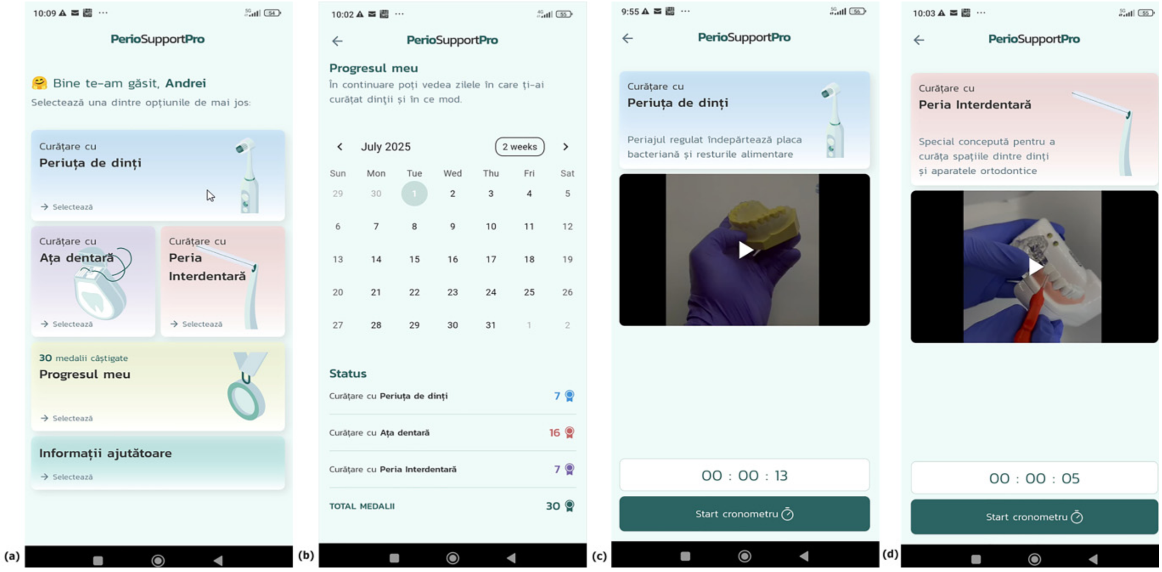

Supplementary material – Figure S1. The second version of *PerioSupportPro* app. (a). App interface at first view, **(b).** Gamification rewards – medals, **(c).** Interface of video module on tooth brushing technique, **(d).** Interface of video module on interdental cleaning technique.
